# The Influence of Selected Process Parameters on the Efficiency of the Process of Gas Nitriding of AISI 1085 Steel

**DOI:** 10.3390/ma17112600

**Published:** 2024-05-28

**Authors:** Tadeusz Frączek, Rafał Prusak, Jerzy Michalski, Zbigniew Skuza, Marzena Ogórek

**Affiliations:** 1 Department of Materials Engineering, Faculty of Production Engineering and Materials Technology, Czestochowa University of Technology, 42-201 Czestochowa, Poland; tadeusz.fraczek@pcz.pl; 2 Department of Production, Faculty of Production Engineering and Materials Technology, Czestochowa University of Technology, 42-201 Czestochowa, Poland; rafal.prusak@pcz.pl (R.P.); zbigniew.skuza@pcz.pl (Z.S.); 3 Łukasiewicz Research Network—Warsaw Institute of Technology, 01-796 Warszawa, Poland; jerzymichalski987@gmail.com

**Keywords:** gas nitriding, kinetics and efficiency of the process, chemical reactor, thermo-gravimetric measurement

## Abstract

The main aim of the manuscript was to investigate the impact of modifying the parameters of the gas nitriding process of samples made from AISI 1085 steel on the course and results of the process carried out in a chemical reactor allowing for thermogravimetric measurements. The tested steel was subjected in a chemical reactor to the process of gas nitriding in the temperature range of 490–580 °C, using different sample heating rates (in the range of 1–25 °C/min) and various mixtures of nitriding gases (pure NH_3_, or NH_3_ with the addition of H_2_ or N_2_). To assess the impact of the tested process parameters on its efficiency, the thickness of the nitrided layers produced, the change in sample mass, the structure of the phases produced, the phase composition and the microhardness were examined. For the research methodology used, it was found that reducing the amount of NH_3_ and/or using H_2_ or N_2_ admixtures adversely affects the thickness of the nitride layers produced. At the same time, the use of a lower maximum process temperature with the same gas mixture resulted in a significant difference in the thickness of the layers. It was also found that the use of pure NH_3_ or a gas mixture (NH_3_ + H_2_) with higher NH_3_ contents resulted in higher surface microhardnesses of the samples and that for these samples, the hardness increased to a greater depth.

## 1. Introduction

Broadly understood progress requires maintaining an appropriate pace of development of science and generally understood technological thought. The requirements for modern materials are increasingly high, and they are expected to meet more and more restrictive parameters and—taking into account the strong contemporary emphasis on ecological aspects—display increasingly high durability and compliance with the assumptions of sustainable development. In many applications, material parameters such as abrasion or corrosion resistance are of key importance for materials, and they depend on the properties of the surface layers produced.

Nitriding is still one of the fastest developing surface treatment methods. The development of this type of thermo-chemical treatment is aimed at inventing repeatable technologies that allow for obtaining optimal properties for a specific group of metallic materials in industrial conditions. An important element is also the reduction in process costs, which is related to the partial replacement of aggressive and environmentally harmful NH_3_ with cheaper and more environmentally friendly gases such as N_2_ or H_2_.

The first works on nitriding were written in the 19th century, but interest in this process increased only in the early 20th century, both from the point of view of basic and utilitarian research [1,2,3]. Currently, practical applications include gas nitriding and nitriding under glow discharge conditions. Among the mentioned nitriding methods, gas nitriding, which has been developed for over 100 years, is still the most commonly used method. This method allows one to improve the functional properties of the material (surface hardness [4], corrosion resistance [5], wear resistance [6]), all with small changes in the dimensions of the nitrided elements [7].

The main aim of this manuscript was to investigate the impact of modifying the parameters of the gas nitriding process of samples made from AISI 1085 steel on the course and results of the process carried out in a chemical reactor allowing for thermogravimetric measurements. This article presents the results of research on the assessment of the effectiveness of gas nitriding of AISI 1085 steel samples at various process parameters, such as the process duration, maximum process temperature, heating rate and the composition of the gas mixture used during the process. The thickness of the nitrided layers produced, the change in sample mass, the structure of the final phases, their phase composition and their microhardness were examined after the nitriding processes. A significant impact of the use of different process parameters on the layer thickness was found.

AISI 1085 is a structural spring steel intended for the production of friction discs, springs and other machine parts that must be characterized by high strength, flexibility and resistance to abrasive wear. AISI 1085 carbon steel finds various applications where its specific properties, such as high strength and wear resistance, are desirable. Some of the common applications of 1085 carbon steel are as follows: knives and blades, hand tools, springs, agricultural implements, industrial machine parts, cutlery and utensils, tooling and dies.

Gas nitriding (GN) is a thermochemical treatment used to improve selected properties of the nitrided material [3]. The nitriding process takes place at the interface between the gas (NH_3_) and the surface of the workpiece, adsorbing atomic nitrogen on the surface and diffusing this nitrogen into the material [8]. The diffusion of atomic nitrogen leads to the formation of a surface layer whose properties are different from those of the base material. The parameters of this layer, i.e., its thickness and phase structure, depend on the nitrided steel grade as well as the nitriding conditions. The basic parameters of the nitriding process are as follows: temperature T, nitrogen potential of the atmosphere NP and time t. The nitrogen layer consists of a complex zone, including, among other substances, iron nitrides ε and γ′ and a diffusion zone with precipitations of γ′ nitrides. A smaller share of the ε phase improves wear resistance, while increasing the share of this phase (with limited porosity) contributes to an increase in corrosion resistance [9].

The nitriding process can be carried out by means of single-stage or two-stage nitriding, using various gas mixture compositions.

One of the most common gas nitriding methods is the single-stage process [10] which, unfortunately, provides limited control of the process and limited impact on the final results. This process uses ammonia, the consumption level of which depends on the expected phase composition of nitrides (the highest ammonia consumption occurs in the ε + γ′ + α variant, and the lowest in the α variant). The nitriding process using only NH_3_ requires a fairly simple and cheap treatment system. The operation of this system does not require advanced operator skills, which is its advantage and reduces the costs of the process [4,5].

A more modern process is regulated gas nitriding using a binary atmosphere of ammonia and dissociated ammonia (NH_3_ + NH_3dis._) [6,7]. This method enables the use of appropriate gas mixtures (dilution of NH_3_ with NH_3dis._) that shape the chemical environment of the process. This allows the process to be controlled in such a way that it is possible to have a greater impact on the phase composition and thickness of the nitrided layer. Moreover, in this process, the inflow of atomic nitrogen onto the surface of the material is limited, which limits the growth of the ε or ε and γ′ layer (which is of great importance due to unfavorable porosity and brittleness). However, this process has two basic disadvantages from an economic point of view. The first is high NH_3_ consumption. The second necessity is to install a dissociator for the production of NH_3_dis. Since the dissociator is a hydrogen-producing device, some users are reluctant to apply it due to the possibility of creating an explosive mixture of hydrogen and oxygen [8,9,10].

Another commonly used process is regulated two-stage gas nitriding—NITREG [4,5,6]. This modern method of thermo-chemical treatment produces layers with a small white zone thanks to the appropriate selection of the chemical composition of the nitriding atmosphere. This eliminates the need for grinding after nitriding, which improves the cost-effectiveness of the method.

Another variant of the process involves the use of a binary atmosphere of NH_3_+N_2_, in which NH_3dis._ is replaced by molecular nitrogen N_2_. This eliminates the need to use an expensive dissociator [11]. This process ensures a large impact on the final effect of the process (phase structure of the nitrided layer) with lower NH_3_ consumption. However, the main disadvantages of this process are higher nitrogen consumption and difficulties in controlling the process kinetics due to its non-equilibrium nature [12]. The development of the nitriding process for specific details requires more tests than the preparation of equilibrium processes, i.e., using NH_3_ or NH_3_ + NH_3dis._, and therefore it is undoubtedly a time-consuming process [13,14,15].

It should be emphasized that nitrided layers with the required phase composition can be formed during Nitrovac vacuum nitriding [16] and ion nitriding [17,18,19]. The basic advantages of these processes, from an economic and ecological point of view, include low consumption and low gas emissions. The main disadvantage of these processes is the higher costs resulting primarily from the costs of the required installation [20]. Ion nitriding is currently one of the most cost-effective and rapidly developing technological processes of thermo-chemical treatment. The attractiveness of this method is directly related to the amount of electric energy consumed per unit of mass of the processed material. The glow discharge used during the ion nitriding process causes heating only of the charge (without heating the entire working chamber of the furnace). This method of heating allows the energy consumption of the process to be reduced by 30–50% compared to conventional thermo-chemical treatments. Currently, the fastest developing surface treatment methods include nitriding, heat and thermo-chemical treatment in vacuum, low-temperature plasma, and plasma and laser methods [21]. It should be mentioned that research conducted at the Czestochowa University of Technology indicates that one of the methods of further reducing costs is the use of active screening [17,21,22].

Another nitriding method that uses only NH_3_ is ZeroFlow [12,23,24]. This method allows one to control the nitrogen potential (Np) as a result of the regulated NH_3_ supply. As research has shown, despite using only NH_3_, this method allows for shaping the phase structure of the layer with the same precision as in processes using two-component atmospheres, using fewer resources and producing lower gas emissions. The ZeroFlow nitriding system is simpler than those used for two-stage nitriding processes and those using a binary atmosphere of NH_3_+N_2_ [25,26,27].

## 2. Research and Methodology

**Figure 1:**
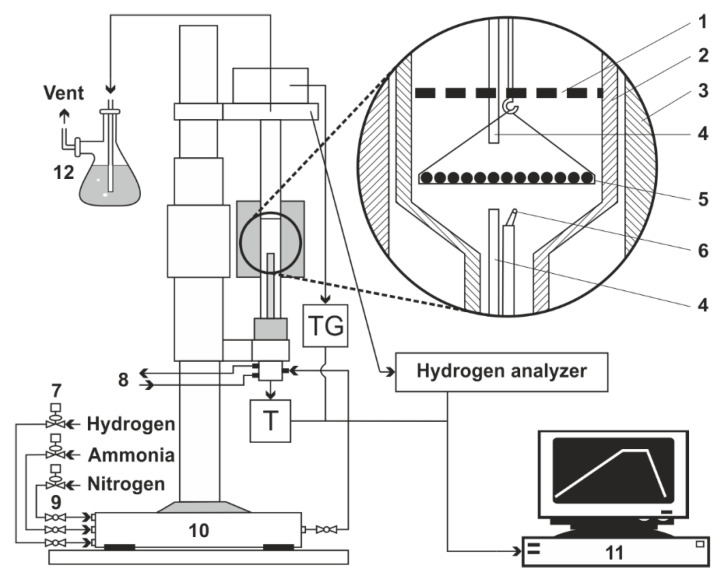
Diagram of a test bench with a thermocouple: 1—shutter; 2—reactor wall; 3—reactor furnace; 4—gas phase sampling point; 5—sample holder with single layer of grains; 6—thermocouple; 7—electronic flowmeters;8—cooling water (closed circuit); 9—ball valve; 10—gas mixer; 11—process control computer; 12—scrubber (distilled water) [28].

As part of this research work, AISI 1085 alloy steel (Table 1) was heated to selected nitriding temperatures of 490–580 °C. The process was carried out in a chemical reactor with precise measurement of the change in the mass of the samples being nitrided (so-called “thermobalance”, Figure 1), enabling the monitoring and recording of the change in sample mass during the nitriding, with a resolution of 50 µg. The reactor is a computer-controlled device consisting of an electrically heated cylindrical chamber in which a sample of a solid substance (lying directly or in a crucible on the pan of a mass change measurement system) is weighed [28]. The device allows one to record changes in basic parameters during the process: sample weight, temperature in the chamber, hydrogen share. The scheme of the test stand is shown in Figure 1. The devices is described in more detail in. 

Eight sets of samples were tested. For samples S1–S5 (Table 2), an identical maximum process temperature of 580 °C was used. The differences involved the composition of the gas mixture used and, in the case of sample S5, the duration of the process (twice as long). Moreover, nitriding variants were used for samples S6–S8, differing in the maximum temperature and the gas mixture composition. The purpose of selecting these nitriding variants was to verify the influence of process parameters on the results obtained: changes in the mass of the samples and the thickness and phase composition of the iron nitride layer (white layer).

Various nitriding methods were used in this work, i.e., the 1-stage method using the NH_3_ atmosphere and the 2-stage method using the binary atmosphere of NH_3_ + N_2_ or NH_3_ + N_2_.

Samples in the form of balls with a diameter of 3 mm made from AISI 1085 steel were used for testing. The chemical composition of the steel and the diameters of the spherical samples are given in Table 1. The parameters of the nitriding processes are shown in Table 2.

This research work used an X-ray diffractometer, the Seifert 3003TT, allowing the identification of individual phases forming the nitrided layer, and the basic parameters of diffractometer measurement are presented in Table 3.

The iron nitride layer thickness assessment method developed and used in this paper has been described elsewhere [25].

## 3. Results and Discussion

**Figure 2:**
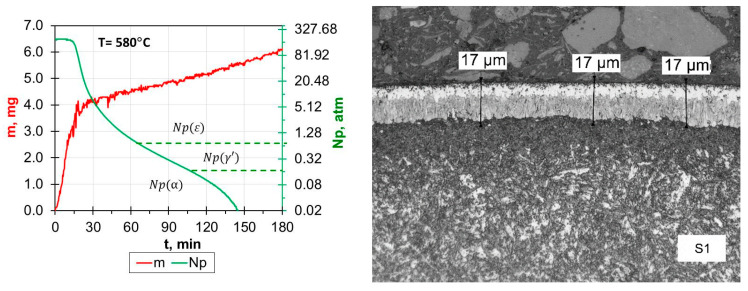

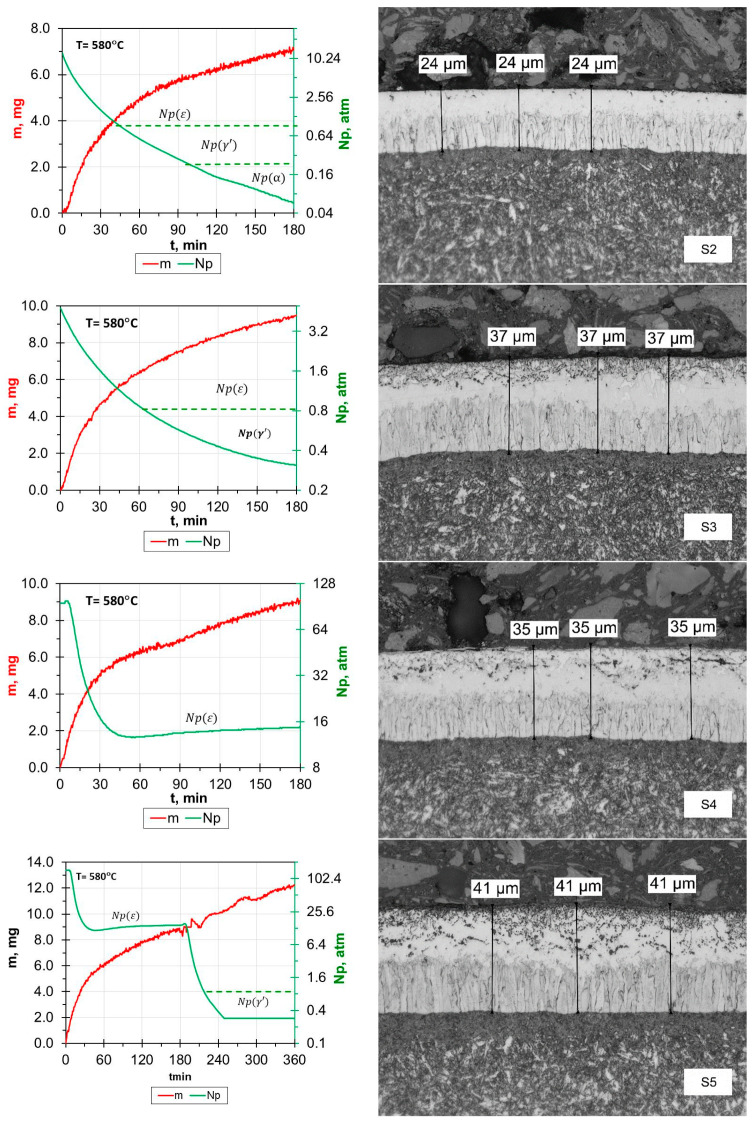
Process proceeding characteristics: N_P_—nitrogen potential; m—weight gain; Np (α), Np(γ’), Np(ε)—value of the nitrogen potential from the stability area of phase α, γ and ε, respectively, and the microstructure of nitrided samples 1–5.

In the case of samples from the S1–S5 group, the iron nitride layers that formed as a result of the nitriding process were of uniform thickness. The presence of ε and γ′ iron nitrides was confirmed by both the results of phase composition analysis (X-ray drawing) and observations of the obtained microstructures of the surface layers (Figure 2).

Moreover, the boundary between the iron nitride layer and the substrate was clearly visible (Figure 2). The thickness of the iron nitride layers produced (g_mp_) changed with the changes in the parameters used and ranged from 19 μm for sample S1 to 41 μm for sample S5. This clearly indicates that reducing the amount of NH_3_ and/or diluting the inlet atmosphere with H_2_ reduces the thickness of the iron nitride layers (white layers). The value of the nitrogen potential (Np) obtained during the initial 3 h of nitriding only in NH_3_ (sample S5) favored the formation of a layer of iron nitrides (white layer) with a dominant share of the ε phase with a thickness of approx. 35 µm (similar to the S4 process). During the next 3 h, the growth rate of the iron nitride layer was reduced as a result of a significant decrease in the value of the nitrogen potential, which resulted in a phase transition of the ε zone into the γ’ phase. Extending the process time from 3 h (S4 process) to 6 h (S5 process) resulted in obtaining the thickest nitrided layer (41 μm).

**Figure 3:**
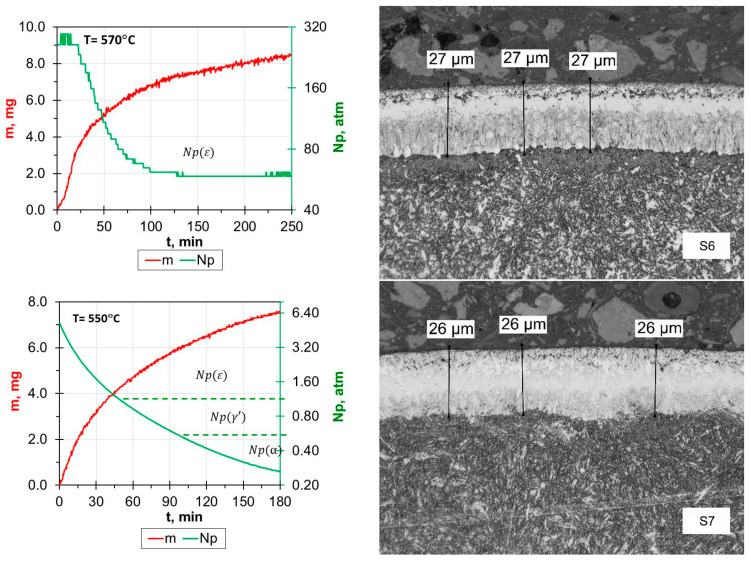

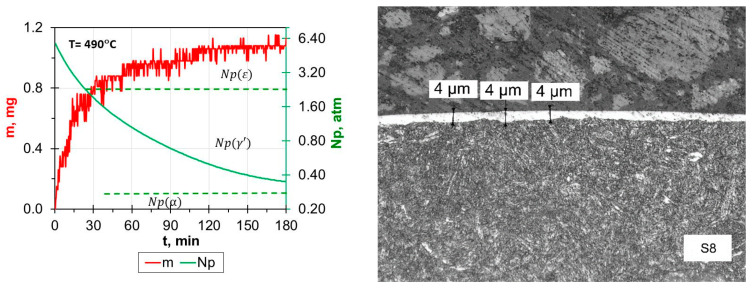
Process proceeding characteristics: Np—nitrogen potential; m—weight gain; Np (α), Np(γ’), Np(ε)—value of the nitrogen potential from the stability area of phase α, γ and ε, respectively, and the microstructure of nitrided samples 6–8.

The analysis of the effects of the nitriding process for samples from the S6–S8 group allowed the conclusion that—similarly to the previous group—the nitride layers produced had a uniform thickness and the boundary between the produced layer and the substrate was clearly visible (Figure 3). The iron nitride layers on samples S6 and S7 had almost identical thicknesses, which indicates that neither the difference of 20 °C with respect to the maximum process temperature nor the differences in the heating rate (10 °C/min and 25 °C/min, respectively) influenced the thickness of the layers. What is striking—in the context of the layer thickness—is the virtually invisible effect of using different gas mixtures (for S6—120 mL/min NH_3_ and 180 mL/min N_2_, for S7—190 mL/min NH_3_ and 10 mL/min H_2_).

**Figure 4:**
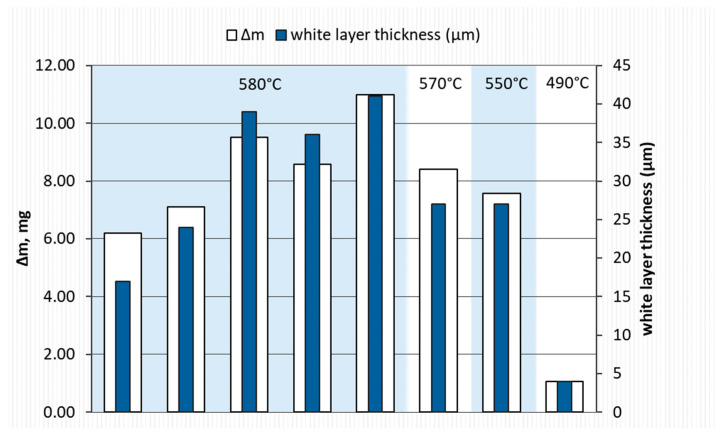

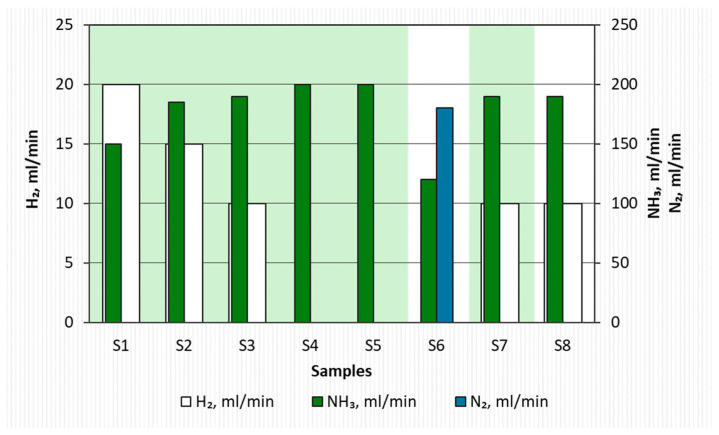
Weight gain and layer thickness depending on the process parameters.

Figure 4 shows data on the influence of the gas mixture used on the thickness of the nitride layer produced and on the weight gain of the samples (total increase in sample weight as a result of the nitriding process). It can be clearly observed that the largest thicknesses of nitrided layers were obtained for samples S3–S5, for which the process was carried out either only in NH_3_ or with a small addition of H_2_. Moreover, differences in the process results for samples S3 and S7 show that the use of a lower process temperature (580 °C and 550 °C, respectively)—with the same gas mixture—resulted in a difference in the thickness of the produced layers of approx. 10 μm in favor of sample S3. Further lowering of the process temperature (sample S8) led to an expected significant reduction in the thickness of the nitride layer, despite the use of the same gas mixture. It is also significant that even though the process time is extended at a lower temperature, it leads to the formation of layers of much smaller thickness. This is consistent with the so-called diffusion laws, Fick’s laws, according to which the decisive factor influencing the diffusion rate is the process temperature.

The change in the mass of the samples was obviously correlated with the thickness of the nitride layers produced. The highest mass increase was found for sample S5 (Δm = 10.98 mg), while the smallest was observed for sample S8 (Δm = 1.06 mg). This is primarily due to the much lower process temperature.

**Figure 5:**
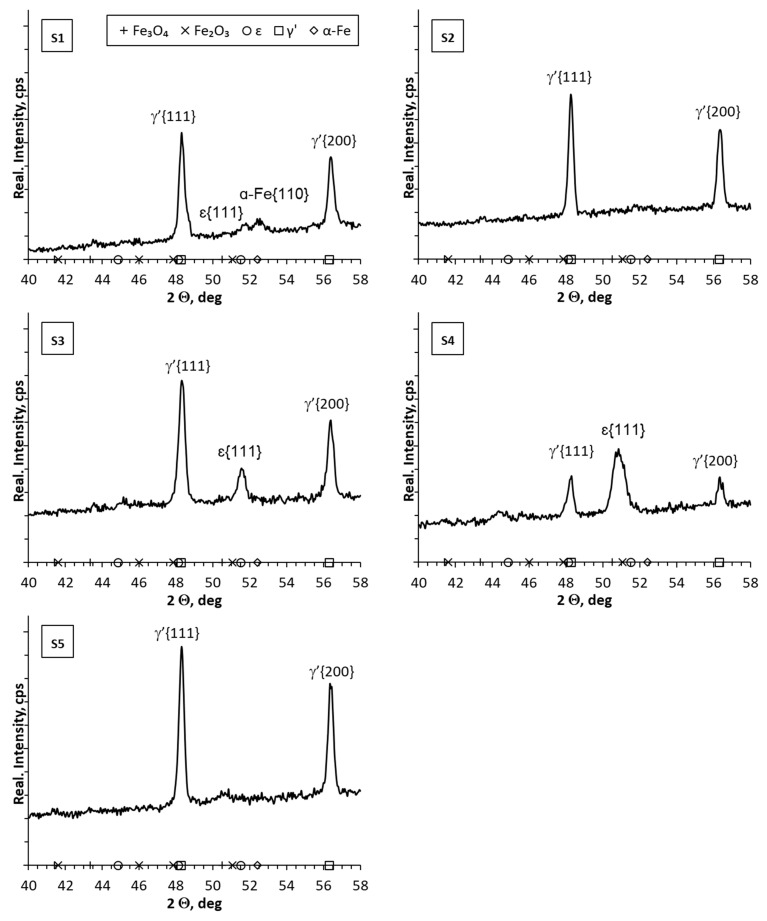
X-ray analysis results for samples 1–5.

**Figure 6:**
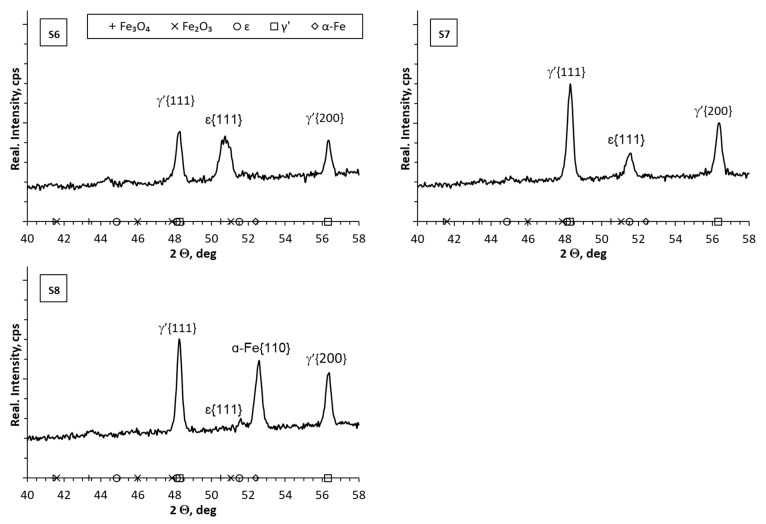
X-ray analysis results for samples 6–8.

In the diffractograms from the surface of samples S1–S5 (Figure 5), lines characteristic of the γ’ phase were identified, and in samples S3 and S4 lines characteristic of the ε phase were also found. In sample S1, a characteristic line of α-Fe was identified, in addition to the lines characteristic of nitride phases. In the case of samples S1, S2, S3 and S5, the values of the nitrogen potential in the initial stage of the nitriding process corresponded to the stability of the ε phase. Then, the nitrogen potential was lowered to the value corresponding to the stability of the γ’ phase, and then—in the S1 and S2 processes—the nitrogen potential was lowered to the value corresponding to the stability of the α phase. On the other hand, in the case of sample S4, the nitrogen potential assumed values in the area of stability of phase ε during the entire nitriding process. Analyzing the influence of the nitrogen potential on the thickness of the formed iron nitride layer (white layer), it can be concluded that the thinnest layers were obtained when the nitrogen potential in the last stage of the process had values corresponding to the stability of the α phase (S1, S2). When the process did not include the stage in which the nitrogen potential assumed values corresponding to the stability of the α phase, the iron nitride layers were thicker (S3, S4, S5). In the case of samples S6–S8 (Figure 6), the differences in the diffractograms were more visible. In addition to the lines characteristic of the γ’ layer, lines characteristic of the ε and α-Fe layers were also identified. The lines appearing in the diffractograms, characteristic of iron oxides (Fe_3_O_4_, Fe_2_O_3_), may be the result of flushing the reactor with nitrogen at too high a temperature. This resulted in the appearance of oxide phases on the surface of the iron nitride layer (white layer). The research methodology will be adjusted in subsequent studies to avoid the occurrence of this type of effect.

**Figure 7:**
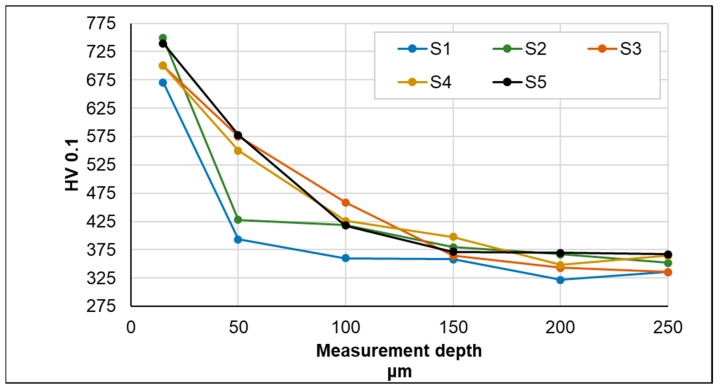
Hardness measurement results for different process parameters (S1–S5).

**Figure 8:**
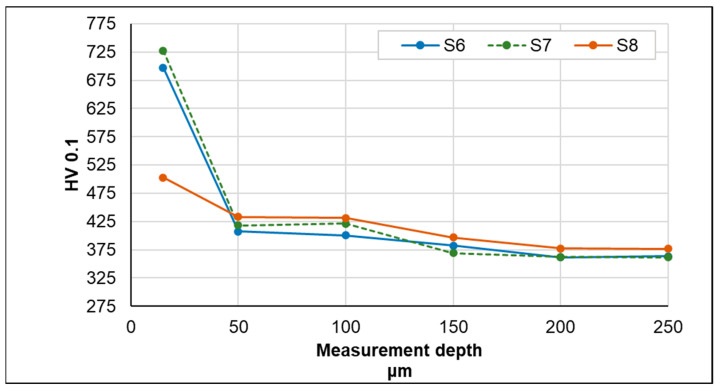
Hardness measurement results for different process parameters (S6–S8).

Testing the hardness of the samples demonstrated the existence of certain differences between the individual nitriding variants (Figure 7 and Figure 8). For samples S1–S5, it can be observed that the use of pure NH_3_ or a gas mixture (NH_3_ + H_2_) with higher NH_3_ contents resulted in higher surface microhardnesses of the tested samples (exceeding 700 HV0.1).

However, the amount of ammonia not only determines the value of hardness, but also the thickness of the layer, as confirmed by the S5 process, in which the process time was twice as long, which resulted in not only high hardness values but also nitrided layers with the greatest thickness (nitride precipitate zone—41 µm + zone diffusion).

With respect to samples S6–S8, it can be observed that the hardness distribution profile in the nitrided layers of samples S6 and S7 is very similar. This clearly indicates that, under the applied process conditions, neither the use of different heating rates nor the addition of N_2_ significantly influenced the obtained effect in terms of hardness. In the case of sample S8, as expected, the reduced process temperature limited the possibility of reactions occurring on the surface of the tested samples, which, in addition to the already mentioned lower thickness of the nitrided layers, was reflected in a slight increase in hardness measured on the surface of the samples. The results obtained in this part of the research allow us to conclude that carrying out nitriding processes in an atmosphere of pure NH_3_ allows for obtaining layers of higher hardness and, additionally, the hardness increases to a greater depth.

## 4. Conclusions

Traditional gas nitriding, usually carried out using a single-component atmosphere (ammonia), is characterized by very limited control over the kinetics of the nitride layer growth, and therefore the formed layer most often consists of ε + γ′ + α zones. The use of various gas mixtures in the tests showed that these mixtures—with the same remaining process parameters—allow for obtaining different values of the nitriding potential, and thus for a more controlled development of individual nitride zones. This is important because, as research by other authors shows, the creation of the ε + γ′ + α layer requires a much higher nitriding potential than the creation of the γ′ + α layer, which reduces the economic and ecological efficiency of the process [4]. Additionally, the controlled development of nitride zones allows one to influence the amount of the ε phase present, a smaller amount of which helps to improve wear resistance, while a larger amount to increased corrosion resistance [5]. The nitriding variant used in the conducted research led to high values of the nitriding potential in the initial phase of the processes and its reduction later. This solution resulted in the saturation of the surface layer with nitrogen and the formation of a thin layer of iron nitrides in the first phase and the expansion of the internal nitriding zone in the second phase of the process [6].

The use of thermogravimetric measurements in the gas nitriding process made it possible to determine the impact of selected process parameters on the tested material. The research results allowed for the following conclusions to be drawn:

- Reducing the amount of NH_3_ by adding H_2_ or N_2_ resulted in obtaining nitrided layers of a lower thickness compared to processes using only NH_3_. For samples S1–S5, for which the process temperature was 580 °C, the highest thicknesses of nitrided layers were found for samples S3–S5, for which NH3 or NH_3_ slightly diluted with H_2_ (from 34 to 41 µm) was used; this effect was particularly noticeable for sample S5, for which the heating time was extended (from 180 min to 360 min). Reducing the amount of ammonia or using larger hydrogen admixtures contributed to obtaining smaller layer thicknesses (S1–S2; 17–24 µm) while maintaining the same process parameters. In the case of the S6–S8 sample group, it was observed that processes carried out using different gas mixtures and different temperatures led to layers of the same thickness. The S6 process was carried out in an atmosphere of 40%NH_3_ + 60%N_2_, while the S7 process was carried out in an atmosphere of 90%NH_3_ + 10%H_2_. The high degree of dilution of ammonia with nitrogen in the S6 process caused a greater reduction in the nitrogen flow onto the nitrided surface than dilution with hydrogen in the S7 process. Hence, similar thicknesses of iron nitride layers were obtained on samples S6 and S7.
- In all of the gas nitriding processes, the nitrogen potential was at a high level during the process and decreased in the final stages. For samples S1–S5, it was observed that while maintaining the same process temperature, reducing the amount of NH_3_ led to the potential value decreasing first to levels characteristic of gamma nitrides, and then to the level of alpha nitrides. The use of 200 mL of NH_3_ or 190 mL of NH_3_ resulted in the potential not decreasing to the potential characteristic of α nitrides.
- The analysis of the diffractograms of the tested samples, despite some differences, generally indicates that when the nitrogen potential in the last stage of the process takes values in the α phase stability region, the result is a monophase γ’ layer.
- The highest microhardness value was found for samples subjected to gas nitriding using pure NH_3_ or a gas mixture (NH_3_ + H_2_) with higher NH_3_ concentrations. At the same time, for these samples, the microhardness increased to a greater depth (approx. 50 μm).

## Figures and Tables

**Table 1:** Chemical composition of steel used in the tests.

Grade Steel	ɸ	Element Content in wt. %				
	(mm)	C	Mn	Si	P	S
AISI 1085	3	0.80–0.93	0.70–1	0.07–0.6	≤0.03	≤0.05

**Table 2:** The basic parameters of the nitriding processes.

Sample No.	T (°C)	VT (K/min)	t (min)	NH₃ (mL/min)	H₂ (mL/min)	N₂ (mL/min)
S1	580 °C	25	180	150	20	0
S2				185	15	
S3				190	10	
S4				200	0	
S5			360			
S6	570 °C	10	180	120		180
S7	550 °C	25		190	10	0
S8	490 °C		240			

**Table 3:** Characteristics of basic diffractometer measurements.

Parameters	Value
voltage	30 kV
current	40 mA
step	2θ 0.05°
counting time	5 s
Range of diffraction angles	40–58°

## Data Availability

The data can be accessed from Czestochowa University of Technology.
